# Untargeted lipidomic analysis of plasma from obese women submitted to combined physical exercise

**DOI:** 10.1038/s41598-022-15236-0

**Published:** 2022-07-07

**Authors:** Rocio San Martin, Camila Fernanda Cunha Brandao, Márcia Varella Morandi Junqueira-Franco, Gizela Pedroso Junqueira, Ellen Cristini de Freitas, Flavia Giolo de Carvalho, Caio Henrique Pinke Rodrigues, Audrey Aguesse, Stéphanie Billon-Crossouard, Michel Krempf, Mikaël Croyal, Julio Sergio Marchini

**Affiliations:** 1 Internal Medicine Department, Ribeirão Preto Medical School, University of São Paulo-FMRP USP, Ribeirão Preto, São Paulo Brazil; 2 University State of Minas Gerais, Divinópolis, MG Brazil; 3 Department of Health Sciences, Ribeirão Preto Medical School, University of São Paulo, Ribeirão Preto, São Paulo Brazil; 4 School of Physical Education and Sport of Ribeirão Preto (EEFERP-USP), University of São Paulo, Ribeirão Preto, São Paulo Brazil; 5 Department of Chemistry, School of Philosophy, Sciences and Letters of Ribeirão Preto, University of São Paulo, Ribeirão Preto, São Paulo Brazil; 6 CRNH-Ouest Mass Spectrometry Core Facility, F-44000, Nantes, France; 7 Nantes Université, CHU Nantes, CNRS, Inserm, BioCore, US16, SFR Bonamy, F-44000 Nantes, France; 8 Clinique Bretéché, Groupe Elsan, F-44000, Nantes, France

**Keywords:** Biomarkers, Lipidomics

## Abstract

This study aimed to determine the changes of lipidome in obese women undergoing combined physical exercise training. Fourteen adult women with obesity (mean BMI and age, 33 kg/m^2^ and 34 ± 5 years), were submitted to combined physical training (aerobic and strength exercises, alternately, 55 min at 75–90% of the maximum heart rate, 3 times a week) for 8 weeks. All participants were evaluated before and after the training intervention for lipidome, anthropometric measurements, muscle strength, and maximum oxygen consumption (VO_2_max). Untargeted liquid chromatography-mass spectrometry analyses allowed the identification of 1252 variables, of which 160 were significant (*p* < 0.05), and 61 were identified as molecular species of lipids. Volcano plot analysis revealed LPC(16:0p), LPC(18:0p), LPC(20:2), and arachidonic acid upregulated and PC(38:1p), PC(40:4), PC(40:4p) downregulated after combined physical exercise. From the results of the overall Principal component analysis (PCA), the major finding was SM(d18:1/20:0), arachidonic acid, and PC(40:6) species. Other changes included a reduction in waist circumference (Δ = − 2 cm) (*p* < 0.05), with no weight loss. In conclusion, 8-week of combined exercise training in obese women brought changes in different classes of lipids. This study provides further information to understand the effect of combined physical exercise on lipids related to obesity.

## Introduction

Obesity is considered a global epidemic and its prevalence is increasing at an alarming rate. It is estimated that 2.1 billion people worldwide are obese or overweight, representing nearly 30% of the world's population^1^. Traditionally, obesity is seen as a high energy intake and a sedentary lifestyle, resulting in a positive energy balance that will be stored as energy in the adipose tissue^2, 3^. However, obesity is much more complex, as several internal and external factors contribute to this growing challenge^4, 5^.

Despite the progressive increase in obesity, it is important to emphasize that there are strategies that can prevent the current scenario^6^. Physical exercise has been proposed as an efficient measure for long-term obesity prevention^7^. Therefore, several studies have been carried out to determine the effects of physical exercise on plasma lipidome in people with obesity, and/or abnormalities in energetic substrates used during physical exercise^8–12^. Physical exercise leads to physiological changes in energy homeostasis, as it depends on changing cellular responses to internal and external stress^13^. In this line, some reports observed that exercise can also affect human metabolism in plasma, which is the most responsive medium to changes in the body^14, 15^.

Through lipidomics, is possible to study and to identify different lipid species^16^ and is extremely relevant for obesity research since is a disease in which lipids accumulate in the adipose tissue^17^. Thus, plasma lipidome analysis allows the evaluation of lipids classes, in a pathological condition like obesity. Recently, studies involving lipidomics and exercises physiology have been conducted^8, 9^. However, the literature is still scarce, and the remaining knowledge gaps associated with lipidomic patterns in obese women submitted to combined physical training are still unclear. Thus, the present study’s hypothesis is to determine the changes in plasma lipidome in obese women submitted to 8 weeks of the combined physical through untargeted lipidomic analysis.

## Methods

### Ethical consideration

The study was performed in agreement with the Declaration of Helsinki principles. Ethical approval was obtained from the Clinical Hospital Research Ethics Committee from the Ribeirão Preto Medical School, University of São Paulo (protocol number: 1387.040/2016) and registered in ClinicalTrials.gov (registration date: 18/04/2017, NTC 03119350). All subjects gave free written informed consent to participate.

### Study protocol and participants

A prospective longitudinal study was conducted on 20 sedentary adult women (body mass index: BMI 33 ± 3 kg/m^2^, 35 ± 6 years old) selected by a convenience sample. Participants were enrolled in the Brandao et al.^18^ study, an exercise-based randomized controlled trial (registration date: 18/04/2017, clinicaltrial.gov: NCT03119350), approved by the Research Ethics Committee of the Clinical Hospital from the Ribeirão Preto Medical School, University of São Paulo (protocol number: 1387.040/2016). Inclusion criteria were: 20–40 years old, BMI of 30–40 kg/m^2^, sedentary (at least 6 months), and regular menses. Participants who reported a history of diabetes, hypertension, dyslipidemia, cancer, and any obesity‐specific treatment (drugs or bariatric surgery) were excluded. During the intervention, we emphasized to all participants to keep constant their food intake. Six participants withdrew (two became ill and four refused to end the study for no specific reason). Therefore, 14 obese women completed the intervention. It was used a healthy control group (n = 12; age = 37 ± 9 years; IMC = 22 ± 2 kg/m^2^; waist circumference = 80 ± 7 cm) to normalize the lipidomic data obtained for relative abundance (%).

### Physical training intervention

The intervention was executed in a Ribeirão Preto School Gym of the University of São Paulo, supervised. The subjects, who completed the intervention, had a mean 80% of participation. The combined physical training (alternating strength and aerobic exercise) consisted of 15 stations of resisted exercises (for all the main muscle groups) for 30 s (at least 10 repetitions per exercise) alternated with 30 s of jogging. The intervention lasted 10 weeks (2 weeks of adaptation of exercises and 8 weeks of physical training), with a frequency of 3 times/week with 55 min/day of duration, the intensity of 75–90% of Maximum heart rate (HRmax), and multiple repetitions (RM) (2 weeks of 75 at 80%, 4 weeks of 80 at 85% and 2 weeks of 85 at 90%). The intensity of training was controlled by a heart frequency meter (Polar®)^18^. Blood samples were collected after 12 h of fasting in tubes with heparin and EDTA, the plasma was separated by centrifugation at 2058*g* for 15 min (4 °C) and stored in a freezer − 80 °C until the analysis is carried out.

### Plasma untargeted lipidomic analysis

All solvents were purchased from Waters Corporation. First, a quality control (QC) sample was prepared from the aliquots of each biological sample (n = 28; 25 µL of each sample) were then grouped and homogenized. This sample pool (QC) was divided into 12 tubes with 30 µL each. Finally, pre-and post-intervention samples (n = 28) were prepared with 30 µL of each sample and the 12 QC samples for lipid extraction^19^. In brief, 270 µL of ice-cold methanol were initially added to defrost plasma samples. Following 10 s vortex, 750 µL of ice-cold methyl-ter-butyl ether (MTBE) was added and the mixture was vortexed for 10 s. Finally, 225 µL of water was added and vortexed for 10 s. The final mixture was centrifuged (10,000*g*; 10 min, 5 °C) and 800 µL of supernatant was transferred to a Liquid chromatography–mass spectrometry (LC–MS) vial to be evaporated to dryness under a nitrogen stream (room temperature). Dried samples were reconstituted with 200 µL mixture of acetonitrile/isopropanol/water (65/30/5, v/v/v).

Lipid analysis was performed using liquid chromatography coupled to the spectrometry of high-resolution masses (LC-HRMS® UPLCTM Waters and HRMS SynaptTM G2 HRMS Q-TOF) equipped with electrospray ionization (ESI) interface operating in positive ionization, and an Acquity H-Class device® UPLCTM device (Waters Corporation, Milford, MA, USA)^20^. A quality assurance sample (QA) was prepared by pooling 5 µL from each vial. This QA sample was injected throughout the analytical batch for correction and normalization purposes. Samples were randomized and injected (5 µL) altogether with QA extracts onto a reversed-phased LC column. Lipids were eluted. The full-HRMS mode was applied for lipid detection (mass-to-charge ratio (m/z) range 200–1200) at a mass resolution of 25,000 full-widths at half maximum (continuum mode). The ionization settings were as follows: capillary voltage, + 2 kV; cone voltage, 30 V; desolvation gas (N_2_) flow rate, 900 L/h; desolvation gas/source temperatures, 550/120 °C. Leucine enkephalin solution at 2 µg/mL (50% acetonitrile) was infused at a constant flow rate of 10 µL/min in the lockspray channel, allowing for correction of the measured m/z throughout the batch (theoretical m/z 556.2771). Data acquisition was achieved using MassLynxTM software version 4.1 (Waters Corporation)^20^. Sphingolipids species annotated by molecular composition were denoted as <lipid class>  <d> <total number of C in the long-chain base> <total number of double bonds in the long-chain base>/<total number of C in FA moiety> <total number of double bonds in FA moiety> (e.g. Cer d18:1/20:0), the “d” designations used in shorthand notation of sphingolipids refer to 1,3 dihydroxy^21^.

### Statistical analysis

Data normality was verified by the Shapiro–Wilk test and later analyzed by Student's t-test or Wilcoxon, to compare the moments before and after the intervention. The descriptive statistics of the data consisted of mean values ​​and standard error of the mean of both evaluation moments (pre-and post-intervention). The level of significance was determined at *p* < 0.05. Delta (Δ),) in percentage (%) was calculated as follows: (delta) (%) = (final-initial/initial) × 100. A boxplot was used for graphically depicting the relative quantification of Arachidonic acid (n = 14), SM(d18:1/20:0) (n = 11) and PC(40:6) n = 11 through their quartiles. The rectangle spans from the first quartile (lower edge) to the third quartile (upper edge). The segment inside shows the median (with the box divided into 2 equal parts). Lines extending vertically from the box (whiskers) indicate the minimum and maximum values. Univariate statistical analyses were performed using Prism v.9 (Graph Pad, San Diego, California, USA). Multivariate data analyses using PCA and Heatmap were performed using MetaboAnalyst 5.0 using “Euclidean” as the clustering distance and “ward.D” as the clustering method.

## Results

### Study sample characteristics

Table 1 lists the clinical characteristics of the 14 obese women (34 ± 5 years and BMI of 34 ± 3 kg/m^2^) who agreed voluntarily to be evaluated before starting the combined physical exercise. Following 8 weeks of the intervention, body weight and BMI did not change (*p* > 0.05). However, there was a decrease of 2% in waist circumference (*p* < 0.05). Aerobic performance increased 6% of VO_2_max (*p* < 0.05). Strength performance (RM) by bench press was increased by 16% and squat by 89% (*p* < 0.05) (Table 1).

**Table 1:** Anthropometric and physical performance data of obese women before and after combined physical exercise (training).

Variables	Pre-training (*n* = 14)	Post-training (*n* = 14)	*p* value
Age (years)	34 ± 1	–	–
Weight (kg)	86 ± 2	87 ± 3	0.15
Height (m)	1.62 ± 1	–	–
BMI (kg/m^2^)	33 ± 1	33 ± 1	0.20
Waist circumference (cm)	93 ± 2	91 ± 2	0.01
VO_2_max (ml/kg/min)	36 ± 1	38 ± 1	0.02
RM bench press (kg)	30 ± 2	35 ± 3	0.04
RM squat (kg)	36 ± 6	68 ± 5	0.00

Data expressed as mean ± standard error of the mean. BMI body mass index, VO_2_max maximum oxygen consumption, RM Bench press upper limb strength, RM Squat lower limb strength.

### Total lipid identifications by lipid category

LC-HRMS analyses allowed the identification of 1252 variables, of which 160 were significant (*p* < 0.05), and 61 were identified as molecular species of lipids, belonging to major lipid families. Supplementary Table S1 shows the results by showing the relative abundance (%) after physical training of the assigned lipid class.

Thus, those lipids that had a greater decrease were further categorized into phosphatidylcholines (PC), as: PC (36:0) iso1, PC (36:5p), and PC (42:1p), then phosphatidylethanolamine (PE), PE (40:4p), PE (36:5), PE (38:3p), and triacylglycerides (TG) as TG(54:8), TG(56:9) and TG(60:11) (Supplementary Table S1).

On the other hand, those who had a major increase were represented by lysophosphatidylcholines (LPC), as LPC (16:0p); LPC (18:0p) and LPC (20:2), other lipid species of triacylglycerides (TG) (29%) as TG (48:0) and TG (50:0) and finally SM(d18:1/26:1), and arachidonic acid (ARA) (Supplementary Table S1).

### Multivariate lipidomic profiling

**Figure 1:**
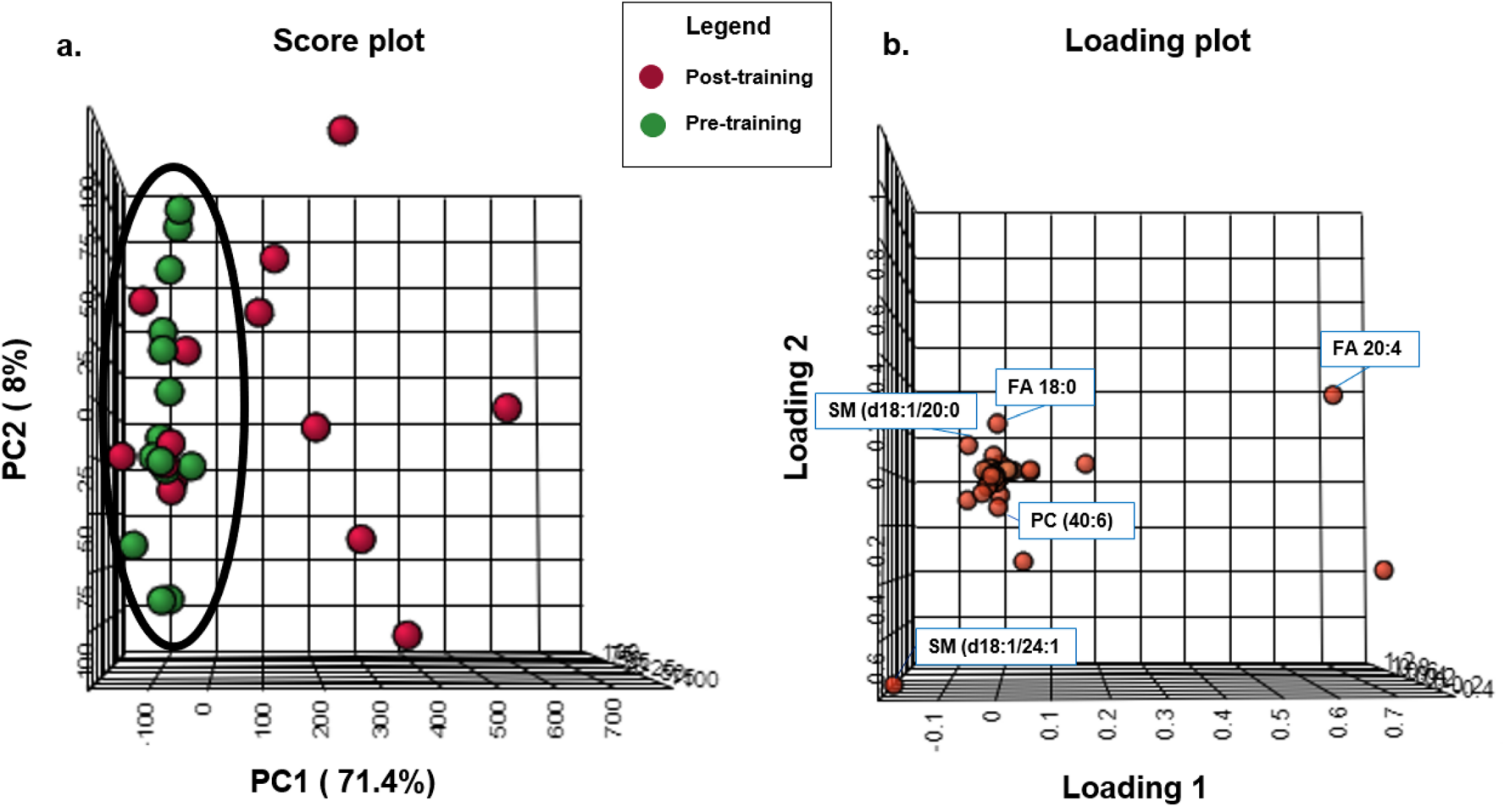
Principal component analysis (PCA). Score plot (**a**) and loading plot (**b**) using the first two principal components obtained from obtained from obese women (n = 14) submitted to combined physical exercise in two moments, pre-training (green) and post-training group (red).

**Figure 2:**
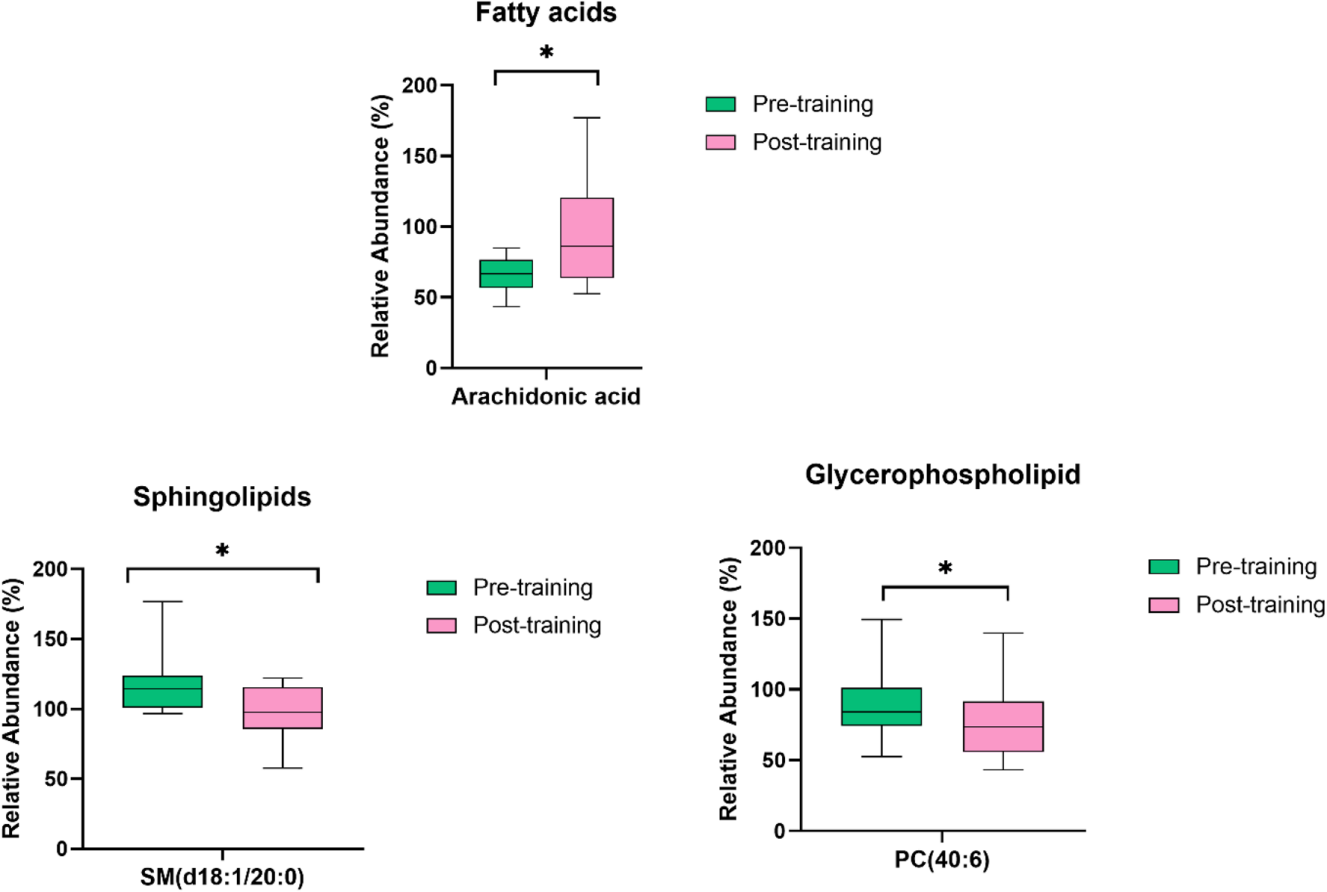
Boxplot of the 3 significantly altered lipids sorted by lipid class with significant differences (*p < 0.05). Green represents the pre-training, and pink represents the post-training intervention. The y-axis is the relative abundance (%). The x-axis corresponds to is each lipid species.

In this study, we applied principal component analysis (PCA) to obtain the data visualization. This analysis provides a resizing of the data, rewriting it in a new set of axes called principal components (PC). The advantage of using PCA is being able to observe the natural organization of the data, which otherwise would not be possible due to the large number of dimensions. PCA on 14 samples and 160 lipids resulted in a model with 2 components, which explained 81% of the total variation. The principal components, especially the first principal component (PC1) and the second principal component (PC2), were drawn in a score plot and any clustering tendency could be observed (Fig. 1). The PC1 accounted for 71% of the total variance that analyzed the sample data according to the plasma lipid level, while the PC2 accounted for 79%. Based on the analysis of the results, it was observed that there was a tendency for arachidonic acid FA(20:4), to be influencing in the group after the intervention, and stearic acid FA(18:0), SM(d18:1/24:1), SM(d18:1/20:0), and PC(40:6) to be the considered most important lipidomic species before the intervention with combined physical exercise (Fig. 2). To visualize another perspective of the graph, see the supplementary material (Supplementary Figs. S1, S2).

### Univariate metabolite and lipid analyses

**Figure 3:**
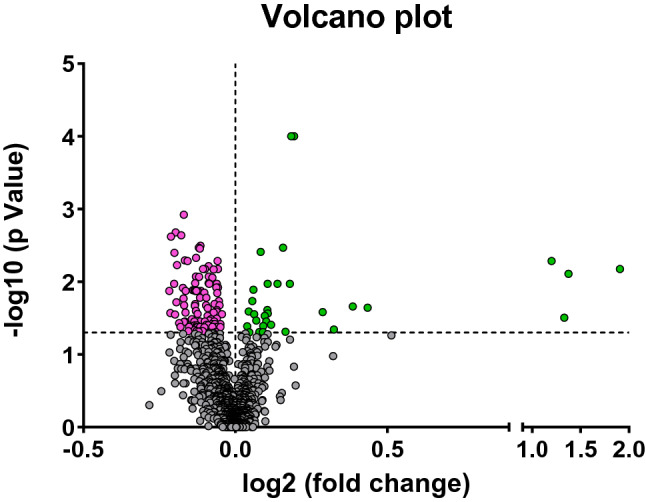
Volcano plot showing the most significant lipid species found by univariate analysis. The volcano plot summarizes both fold-change and t-test criteria for all lipids. It is a scatter-plot of the negative log10-transformed p-values from the t-test plotted against the log2 fold change. Grey values indicate those lipid species are not significant (*p* > 0.05). Negative values (in pink) indicate downregulated lipid species, while positive (in green) values reflect upregulated lipids in women after combined physical exercise (*p* < 0.05).

We next performed a univariate analysis with all the lipids found before and after physical training. After obtaining the raw intensity data, we applied an unpaired Student t-test to each lipid. A volcano plot (Fig. 3) highlights the most significant differences among lipids and the positive or negative fold-change seen in obese women patients after physical training. Significant differences (*p* < 0.05) were the following plasmalogens and lyso-phosphatidylcholines LPC(16:0p), LPC(18:0p), LPC(20:2) respectively, and arachidonic acid found in higher concentrations and phosphatidylcholines PC(40:4) and plasmalogens PC(38:1p), and PC(40:4p) found in lower amounts in obese women after combined physical exercise.

**Figure 4:**
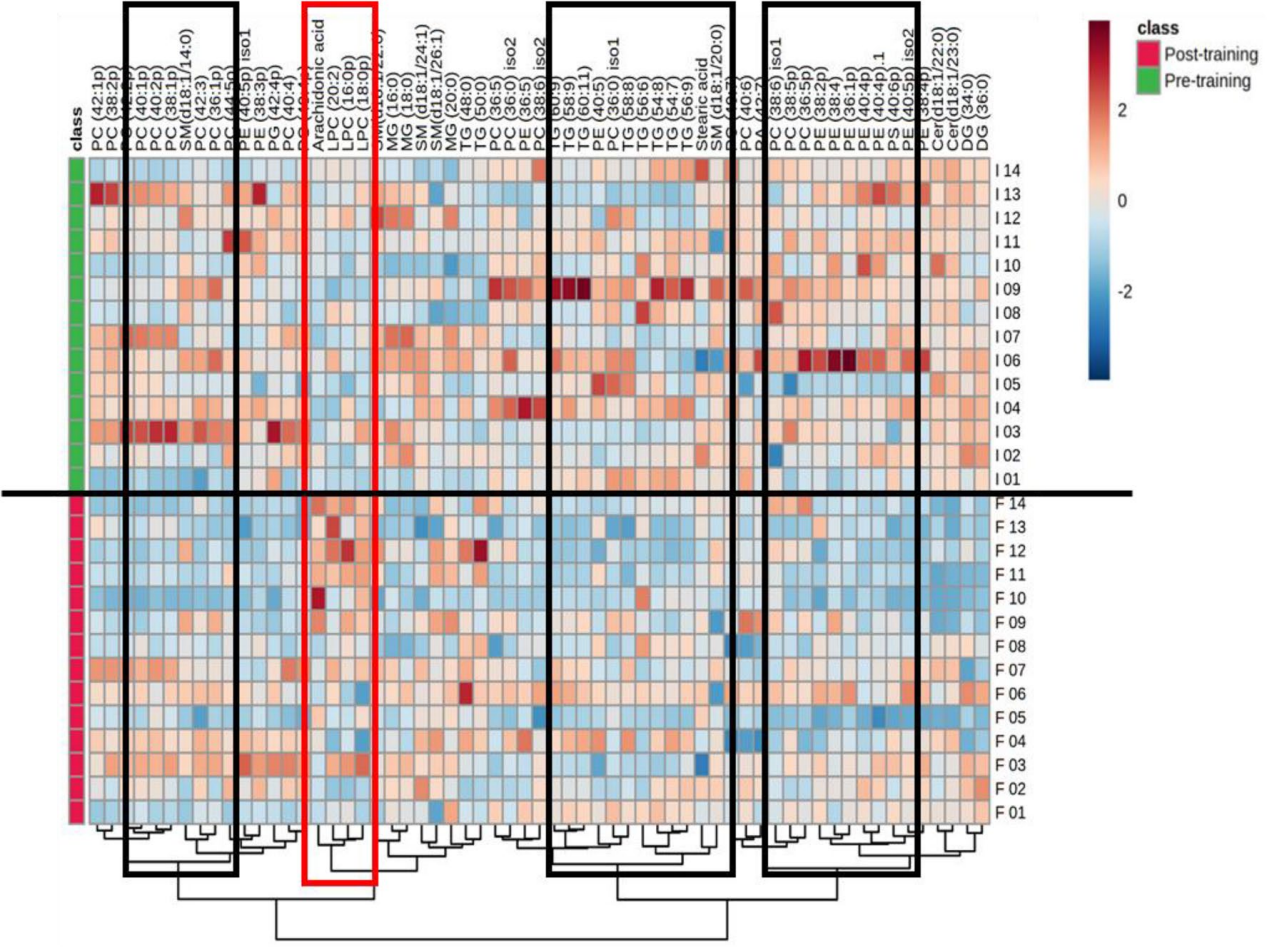
Heat map analysis of hierarchical clustering of plasma lipid species of obese women after combined physical exercise. In this type of analysis, each colored cell on the map corresponds to a concentration value, that is, the unit value corresponding to the measurement performed by mass spectrometry. The range of responses is represented in red and blue. The more intense the red tones, the more positive these values are in relation to the variation of plasma lipids, while the more intense the blue tones, the greater the negative values as a function of the average value of the data. Thus, the 61 main lipid species evaluated for the two groups (pre-training and post-training) are presented and classified by this heat map analysis employing the t-test to retain the most contrasting patterns with 95% confidence. The rectangles were used to demonstrate where there was greater variation before and after training. The clusters are formed using the Euclidean distance clustering method with a Ward algorithm (n = 14). *p ≤ 0.05 for each comparison.

Finally, to summarize our results, we used a heat map (Fig. 4) to display the change of the lipid species before and after combined physical training. The heat map allows the visualization of the different plasma lipids species concentration values according to the pre-or post-training. It is interesting to note, that lipid species as LPC(16:0p,0 LPC(18:0p), PC(20:2), and Arachidonic Acid (shown in the red rectangle), had an increase in their concentrations after the combined physical exercise intervention.

## Discussion

Untargeted plasma lipidomics analyses of this study revealed lipidome changes after combined physical exercise. Specifically, the major changes were focused on fatty acids like arachidonic acid (AA), and stearic acid, phospholipids, glycerophospholipids, and sphingomyelins. To the extent of our knowledge, this is the first study that reveals changes in the lipidome after combined physical exercise in women with obesity.

From the results of the overall PCA, the major finding of this study was the contrast of different lipids species after the intervention. Particularly, SM(d18:1/20:0), arachidonic acid, and PC(40:6) species, were found to influence to separate the groups.

Furthermore, our univariate analysis showed significant differences of LPC(16:0p,) LPC(18:0p), LPC(20:2), and AA that were found in higher concentrations and phosphatidylcholines PC(40:4) and plasmalogens as PC(38:1p), PC(40:4p) found in lower amounts in obese women after combined physical exercise. Other important findings include a decrease in waist circumference, improvement of physical fitness (VO_2_max), and muscle strength.

From a clinical point of view, sphingolipids (SPs), including ceramides, sphingomyelins, can indicate a possible metabolic disorder and cardiovascular diseases^22^. Sphingolipid metabolism is altered in obese subjects because there is an increase of free fatty acids (FFA), which leads to a rise of palmitoyl-CoA following an increase of ceramide production via de novo pathway^23^. A study conducted by Hanamatsu et al.^24^, demonstrated that SM saturated acyl chains (C18:0, 20:0, 22:0, and 24:0) were increased in obese subjects compared to the control group. On the other hand, the LURIC study analyzed the association of various plasma lipid species with mortality^25^, indicating that SM(d18:1/24:1) displayed a positive association with this outcome. Therefore, these results are in agreement with our study, since SM(d18:1/20:0) were found in higher concentrations before the intervention in women with obesity.

On the other hand, it has been shown that physical exercise may alter plasma lipid levels in humans^26^. Hence, these changes may influence sphingolipid as well. For example, some studies showed that after 12 weeks of the supervised exercise-training program and 16 weeks of exercise in overweight/obese subjects may reduce sphingolipids (e.g., C18:0, C20:0, and C24:1) in adults with obesity^27^. Thus, the fact that these sphingomyelins had a decrease after the intervention, could mean that combined physical exercise had a positive effect on women with obesity.

Regarding glycerophospholipids, we found PC(40:6) and PC(40:4) as important molecular species of PCs to be decreased. In contrast, LPC(20:2) was increased after the intervention. Glycerophospholipids have been related to obesity and cardiometabolic diseases^28^. Elevated PC levels have been suggested to increase coronary artery disease and mortality^29^. For example, in a study by Eisinger et al.^30^, was found that PC(40:6) was elevated in mice fed with a high-fat diet (HFD) for 14 weeks and that had an increase in their body weight. Furthermore, Chen et al.^31^ observed that PC(40:6) was positively associated with the incident of metabolic syndrome (MetS). These results could mean that this lipid species could be involved in obesity, which was found in our study. To our knowledge, no publications are naming this lipid as an exercise-induced lipid species.

Circulatory LPCs are derived from the PC of lipoproteins and cell membranes through phospholipase A2^32^. In addition, LPC is generated by endothelial lipase and lecithin cholesterol acyltransferase (LCAT)^33^. LPC is also generated when LDL is oxidatively modified by several mechanisms and LPC has been suggested to be both pro-and anti-atherogenic^34^. In our study, plasma levels of LPC(20:2) increased post-exercise. Indeed, several beneficial effects have been associated with increases in plasma LPC concentrations^35^.

Also, our results revealed changes after the intervention in the following plasmalogens: LPC(16:0p), LPC(18:0p), PC(38:1p), PC(40:4p). Plasmalogens, subclasses of glycerophospholipids are the commonly found ester-carbonyl group in the sn-1position of is substituted by an ether-alkyl or vinyl ether-alkenyl bond^36^. It is known that plasmalogens are involved in the regulation of cellular and systemic lipid homeostasis^37^. Previous reports, observe positive associations of plasmalogens with cardiorespiratory fitness and inverse associations with hypertension, prediabetes, type 2 diabetes mellitus (T2DM), cardiovascular diseases, obesity^37, 38^. Thus, the increase in plasma levels of these plasmalogens in our study may suggest an adaptive response to exercise. Plasmalogens have been proposed to have antioxidant properties, resulting in atheroprotective properties^39^. Although, the physiological functions of plasmalogens are not entirely understood. Some studies are showing an antioxidant role in protecting the cells from oxidative stress against reactive oxygen species^40^.

Concerning the changes in fatty acids found in our research. Stearic acid is an important constituent in human plasma circulation^41^. According to the literature is one of the main fatty acid fuels for aerobic metabolism and has an important role in sports performance^42^. While AA is considered one of the main PUFAs (about 8% of the total) in plasma circulation^41^, Is involved in cellular signaling acting as an inflammatory intermediate, inducing vasodilatation^43^. A previous study has found an inverse relationship of coronary heart disease (CHD) with AA plasma concentrations and a positive association with incident CHD and stearic acid levels^44^. In line with this, in a large prospective cohort, stearic acid was associated with higher type 2 diabetes risk^45^. Mougios et al.^42^ have found a significant decrease in the percentages of stearic acid, at the end of exercise (exercise at 50–55% of maximal aerobic power for 1 h). Although our study did not find a significant difference of stearic acid after the intervention, our exploratory PCA analysis revealed a close association with the pre-intervention moment.

Concerning AA, Contrepois et al.^46^, reports a significant increase of arachidonic acid (AA), within 2 min in response to exercise, which is in line with our results. This increase would be due to the AA’s role in stimulating acute inflammation by controlling local blood flow, vascular permeability, cytokine production, leukocyte chemotaxis, and sensation of pain, suggesting an adaptative response to exercise^47^. Furthermore, it is known that a synthesis of eicosanoids produced from acid arachidonic acid might occur during and after physical activity^43^. The main action of arachidonic acid metabolites is the promotion of acute inflammation in response topical, characterized by the production of inflammatory mediators such as PGE2 and PGI2^43^.

In the context of the clinical variables, our results showed that, after 8 weeks of combined physical training in obese women, there was no reduction in body weight and body composition, however, a decrease in waist circumference was observed. These results are consistent with some authors who showed a reduction in waist circumference after physical training. For example, Kim et al.^48^ observed the effect of a circuit training program on risk factors related to physical health in obese students. As a result of this study, waist measurement and body weight significantly decreased in the circuit training group compared to the control group. Likewise, Martins et al.^49^ found a significant reduction in waist circumference and body weight after 12 weeks (3 times a week) of high-intensity intermittent training (HIIT) in sedentary obese women. Moreover, Jackson et al.^50^, observed that physical training for 8 weeks (3 times a week, 45–90 min per session), with an intensity of 50–90% of VO_2_max, did not lead to weight loss in obese women. These findings are in accordance with what our study observed about a decrease in waist circumference and maintenance of body weight.

Combined physical training also provided positive changes in physical and cardiorespiratory fitness. It has been described in the literature that combined physical training has benefits in cardiorespiratory fitness in people with obesity, regardless of body mass changes^51, 52^.

There are several limitations in this research study. Firstly, it has a small sample size and the lipid profile response to combined physical exercise is not compared with non-obese subjects. Secondly, we do not have information about the food intake of the women with obesity had along with the study. However, we reinforced it to all participants to keep constant dietary intake habits. Our study's advantage is the highly controlled supervised intervention on exercise, including frequency (days per week), duration of each exercise session, and training intensity.

Finally, future work should be the focus on the use of targeted tools to validate these findings to have a quantitative landscape. Thus, additional studies with larger samples are needed to establish potential biomarkers related to lipidome profile and physical exercise.

## Conclusion

Our study suggests that combined physical exercise lasting 8 weeks, promoted increased physical fitness, cardiorespiratory capacity, and reduced waist circumference in obese women. In addition to causing changes in different classes of lipids as sphingolipids, fatty acids, glycerophospholipids, acylglycerols, and plasmalogens. Our study detected lipids that differ after the intervention with physical exercise. These lipids can be the target of studies of molecular mechanisms to understand the underlying biochemical pathways and can serve to improve the monitoring and individualization of training programs in people with obesity.

## Supplementary Information

Supplementary Figure S1.

Supplementary Figure S2.

Supplementary Table S1.

Supplementary Information 1.

Supplementary Information 2.

## Data Availability

All data generated or analysed during this study are included in this published article (and its Supplementary Information files).
