# Supplementary material for: Untargeted lipidomic analysis of plasma from obese women submitted to combined physical exercise
Source: Sci Rep. 2022 Jul 7;12:11541. doi: 10.1038/s41598-022-15236-0 (PMC9263166; doi:10.1038/s41598-022-15236-0)
Supplement: Supplementary file 1 — Supplementary Figure S1. [file 41598_2022_15236_MOESM1_ESM.docx]

Supplementary Figure S1 and legends

**
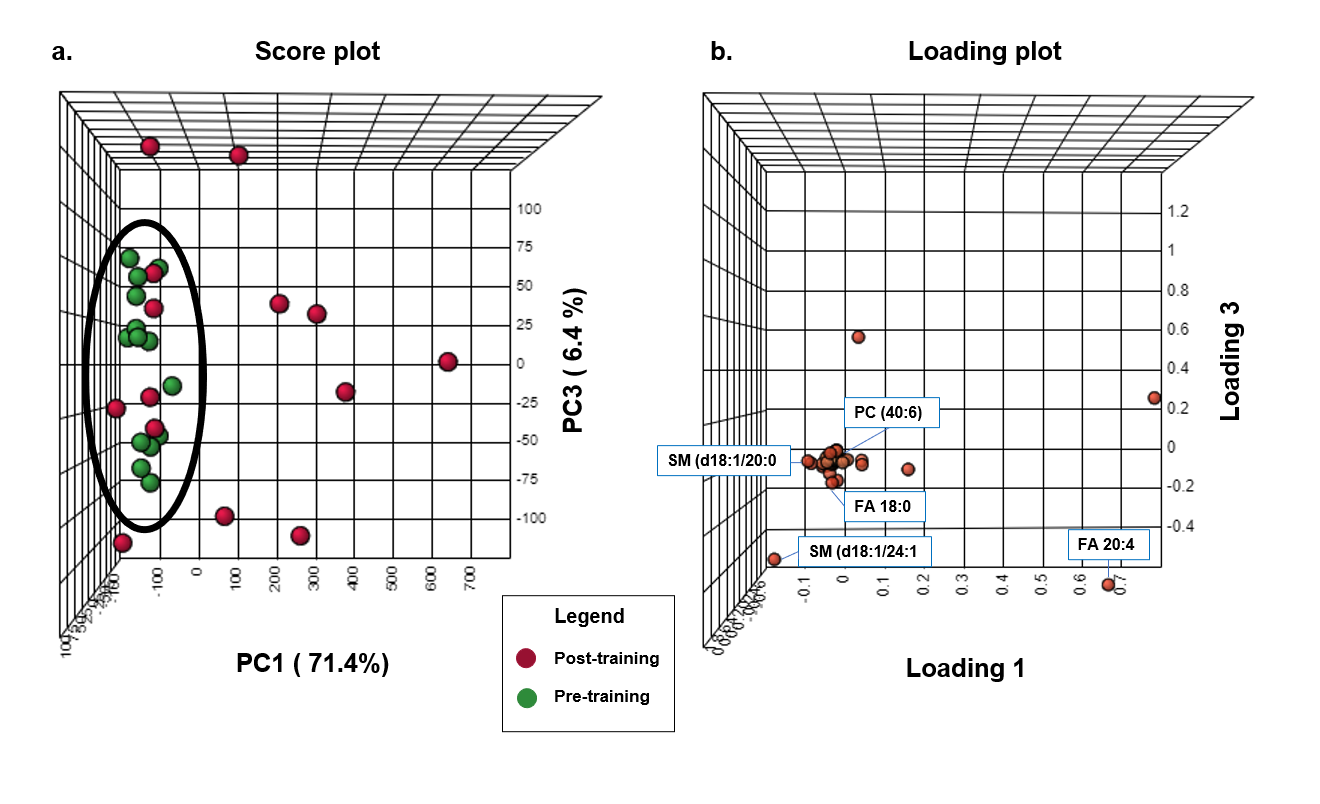
**

**Supplementary Figure S1.** Principal component analysis (PCA). Score plot (a) and loading plot (b) using PC1 and PC3 components obtained from obtained from obese women (n=14) submitted to combined physical exercise in two moments, pre-training (green) and post-training group (red).
