# Supplementary material for: Untargeted lipidomic analysis of plasma from obese women submitted to combined physical exercise
Source: Sci Rep. 2022 Jul 7;12:11541. doi: 10.1038/s41598-022-15236-0 (PMC9263166; doi:10.1038/s41598-022-15236-0)
Supplement: Supplementary file 2 — Supplementary Figure S2. [file 41598_2022_15236_MOESM2_ESM.docx]

Supplementary Figure S2 and legends


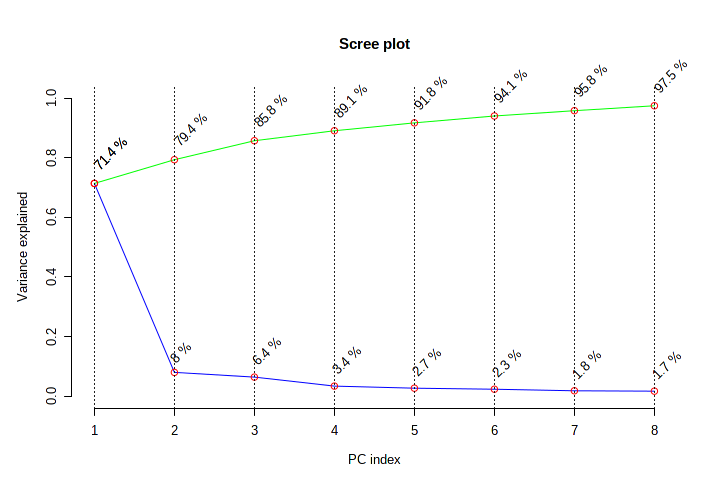


**Supplementary Figure S2.** Cumulative explained variance. The green line on top shows the accumulated variance explained; the blue line underneath shows the variance explained by individual principal component (PC).
