# Supplementary material for: Untargeted lipidomic analysis of plasma from obese women submitted to combined physical exercise
Source: Sci Rep. 2022 Jul 7;12:11541. doi: 10.1038/s41598-022-15236-0 (PMC9263166; doi:10.1038/s41598-022-15236-0)
Supplement: Supplementary file 3 — Supplementary Table S1. [file 41598_2022_15236_MOESM3_ESM.docx]

***Supplementary Information***

Supplementary Table S1 and legends

| **Lipids** | **Symbol** | **Pre** | **Post** | **Δ (*delta*)** | **p** |
| --- | --- | --- | --- | --- | --- |
| **Fatty acids** |  |  |  |  |  |
| Arachidonic acid | FA | 241.5±12.6 | 348.5±36.5 | 44% | 0.01 |
| Stearic acid | FA | 112.1±8.1 | 100.0±4.2 | -11% | 0.04 |
| **Sphingolipids** |  |  |  |  |  |
| Cer(d18:1/23:0) | Cer | 0.8±0.0 | 0.5±0.1 | -35% | 0.04 |
| Cer(d18:1/22:0) | Cer | 1.1±0.0 | 0.6±0.1 | -39% | 0.00 |
| SM(d18:1/26:1) | SM | 0.4±0.0 | 0.5±0.0 | 23% | 0.03 |
| SM(d18:1/24:1) | SM | 276.4±14.5 | 246.7±18.6 | -11% | 0.02 |
| SM(d18:1/22:0) | SM | 5.6±0.4 | 4.9±0.4 | -13% | 0.04 |
| SM (d18:1/20:0) | SM | 93.5±5.2 | 79.3±4.7 | -17% | 0.01 |
| SM(d18:1/14:0) | SM | 22.1±1.5 | 19.2±1.4 | -13% | 0.03 |
| **Glycerolipids** |  |  |  |  |  |
| MG(20:0) | MG | 0.5±0.0 | 0.6±0.0 | 9% | 0.04 |
| MG(18:0) | MG | 2.5±0.1 | 2.2±0.1 | -12% | 0.01 |
| MG(16:0) | MG | 0.8±0.0 | 0.7±0.0 | -12% | 0.01 |
| DG(36:0) | DG | 4.2±0.2 | 2.5±0.5 | -39% | 0.01 |
| DG(34:0) | DG | 5.4±0.2 | 4.5±0.4 | -17% | 0.04 |
| TG(60:11) | TG | 2.6±0.5 | 1.8±0.4 | -30% | 0.05 |
| TG(60:9) | TG | 0.9±0.2 | 0.7±0.1 | -24% | 0.04 |
| TG(58:9) | TG | 14.4±2.3 | 10.2±1.7 | -30% | 0.04 |
| TG(58:8) | TG | 5.4±0.5 | 4.1±0.5 | -25% | 0.02 |
| TG(56:9) | TG | 5.9±1.1 | 4.0±0.7 | -32% | 0.02 |
| TG(56:6) | TG | 58.8±3.6 | 49.4±4.7 | -16% | 0.04 |
| TG(54:8) | TG | 3.5±0.5 | 2.2±0.3 | -37% | 0.01 |
| TG(54:7) | TG | 20.5±2.4 | 15.4±1.9 | -25% | 0.04 |
| TG(50:0) | TG | 4.8±0.4 | 6.6±0.6 | 38% | 0.01 |
| TG(48:0) | TG | 5.8±0.5 | 7.1±0.8 | 23% | 0.05 |
| **Plasmalogens** |  |  |  |  |  |
| LPC(16:0p) | LPC | 1.8±0.1 | 2.2±2.2 | 25% | 0.02 |
| LPC(18:0p) | LPC | 0.4±0.1 | 0.6±0.1 | 44% | 0.01 |
| PC(44:5o) | PC | 1.7±0.2 | 1.2±0.2 | -26% | 0.01 |
| PC(42:2p) | PC | 3.8±0.5 | 2.8±0.4 | -26% | 0.01 |
| PC(42:1p) | PC | 1.2±0.2 | 0.9±0.1 | -27% | 0.02 |
| PC(40:4p) | PC | 0.5±0.0 | 0.4±0.1 | -13% | 0.04 |
| PC(40:2p) | PC | 2.6±0.3 | 2.2±0.3 | -15% | 0.05 |
| PC(40:1p) | PC | 1.2±0.1 | 0.9±0.1 | -24% | 0.1 |
| PC(38:5p) | PC | 1.0±0.1 | 0.8±0.1 | -23% | 0.05 |
| PC(38:2p) | PC | 7.0±0.7 | 5.4±0.6 | -22% | 0.01 |
| PC(38:1p) | PC | 2.7±0.3 | 2.3±0.2 | -14% | 0.02 |
| PC(36:5p) | PC | 1.3±0.2 | 0.9±0.2 | -31% | 0.05 |
| PC(36:1p) | PC | 2.3±0.3 | 1.9±0.2 | -18% | 0.01 |
| PS(40:6p) | PS | 11.5±1.0 | 8.0±0.9 | -30% | 0.03 |
| PG(42:4p) | PG | 2.7±0.2 | 2.0±0.2 | -26% | 0.01 |
| PE(40:5p) iso1 | PE | 0.8±0.1 | 0.6±0.1 | -27% | 0.04 |
| PE(40:5p) iso2 | PE | 3.7±0.3 | 2.5±0.4 | -31% | 0.01 |
| PE(40:4p) | PE | 10.1±1.1 | 5.7±0.7 | -44% | 0.01 |
| PE(40:4p) | PE | 2.2±0.2 | 1.6±0.1 | -26% | 0.01 |
| PE(38:4p) | PE | 49±4.0 | 35.9±4.4 | -27% | 0.04 |
| PE(38:3p) | PE | 12.3±1.5 | 8.0±1.3 | -35% | 0.05 |
| PE(38:2p) | PE | 1.0±0.1 | 0.8±0.1 | -24% | 0.00 |
| PE(36:1p) | PE | 2.2±0.3 | 1.7±0.2 | -25% | 0.03 |
| **Glycerophospholipids** |  |  |  |  |  |
| LPC(20:2) | LPC | 0.6±0.1 | 0.8±0.1 | 20% | 0.03 |
| PC(42:3) | PC | 0.4±0.1 | 0.3±0.1 | -20% | 0.02 |
| PC(40:7) | PC | 7.4±0.4 | 5.6±0.4 | -24% | 0.00 |
| PC(40:6) | PC | 90.3±6.7 | 76.6±7.6 | -15% | 0.01 |
| PC(40:4) | PC | 9.7±0.8 | 8.3±0.9 | -14% | 0.05 |
| PC(38:6) iso1 | PC | 5.6±0.4 | 4.4±0.4 | -20% | 0.03 |
| PC(38:6) iso2 | PC | 30.9±1.8 | 25±1.8 | -19% | 0.02 |
| PC(36:5) | PC | 1.5±0.1 | 1.1±0.1 | -24% | 0.00 |
| PC(36:0) iso1 | PC | 22.2±2.6 | 15.0±1.6 | -32% | 0.00 |
| PC(36:0) iso2 | PC | 19.5±2.4 | 14.4±1.6 | -26% | 0.02 |
| PE(36:5) | PE | 0.9±0.2 | 0.5±0.1 | -39% | 0.03 |
| PE(38:4) | PE | 5.6±0.5 | 4.7±0.3 | -15% | 0.04 |
| PE(40:5) | PE | 1.1±0.1 | 0.8±0.1 | -27% | 0.04 |
| PA(42:7) | PA | 1.7±0.1 | 1.4±0.1 | -18% | 0.02 |
| Supplementary Table S1. Relative abundances (%) of species identified for each lipid class pre- and post-combined physical exercise in obese women (normalized by control). Data expressed as mean±standard error of the mean. BMI: body mass index (P<0.05). Cer: Ceramides. SM: Sphingomyelins. LPC: lysophosphatidylcholine. PC: phosphatidylcholine. PE: phosphatidylethanolamine. PS: phosphatidylserine. PA: phosphatidic acid. PG: glycerophosphoglycerols. MG: monoacylglycerol. DG: diacylglycerol. TG: triacylglycerol. FA: fatty acids. | | | | | |
