# Supplementary material for: Untargeted lipidomic analysis of plasma from obese women submitted to combined physical exercise
Source: Sci Rep. 2022 Jul 7;12:11541. doi: 10.1038/s41598-022-15236-0 (PMC9263166; doi:10.1038/s41598-022-15236-0)
Supplement: Supplementary file 5 — Supplementary Information 2. [file 41598_2022_15236_MOESM5_ESM.pdf]

ClinicalTrials.gov Protocol Registration and Results System (PRS) Receipt
Release Date: April 18, 2017

ClinicalTrials.gov ID: NCT03119350

Study Identification

Unique Protocol ID: Process HCRP: 1.387.040/2016

Brief Title: Mitochondrial Energy Metabolism in Obese Women

Official Title: Mitochondrial Energy Metabolism in Obese Women Undergoing Concurrent Physical Training

Secondary IDs:

Study Status

Record Verification: April 2017

Overall Status: Completed

Study Start: April 1, 2016 [Actual]

Primary Completion: July 1, 2016 [Actual]

Study Completion: September 15, 2016 [Actual]

Sponsor/Collaborators

Sponsor: University of Sao Paulo

Responsible Party: Principal Investigator
Investigator: Julio Sergio Marchini [jmarchini]
Official Title: Principal Investigator
Affiliation: University of Sao Paulo

Collaborators:

Oversight

U.S. FDA-regulated Drug: No

U.S. FDA-regulated Device: No

U.S. FDA IND/IDE: No

Human Subjects Review: Board Status: Approved
Approval Number: 42445614.7.0000.5440
Board Name: Human Research Ethics Committee of Hospital das Clínicas de Ribeirão Preto
Board Affiliation: Faculty of Medicine of Ribeirão Preto, University of São Paulo
Phone: +55 (16)3602-2228
Email: cep@hcrp.usp.br
Address:

Data Monitoring: Yes

FDA Regulated Intervention: Yes

Section 801 Clinical Trial: Yes

Study Description

Brief Summary: Considering that the failure of the treatment of obesity is justified by the multifactorial pathophysiology of this morbidity, the present project has the following hypotheses:

1. The occurrence of obesity is due to the derange,ent of mitochondrial energy metabolism ;
2. The unbalance is therapeutically modified through physical training ;
3. Obesity courses with the break-down in energy metabolism mitochondrial disease associated with systemic inflammatory characteristics that can be corrected through a combined long-term physical training program.

This study have as objective : to analyse changes in mitochondrial function, inflammatory profile, oxidative stress and energy metabolism caused by concurrent physical training in obese women.

Detailed Description: Specific objectives:

Body composition by deuterium oxide; Metabolic rate of resting and oxidation of substrates by indirect calorimetry; Proinflammatory cytokines Anti-inflammatory cytokines Oxidative Stress: Malondialdehyde, Superoxide Dismutase, Glutathione-Peroxidase; Fatty acids: ceramide and palmitate; Mitochondrial respiration and citrate synthase enzyme; Quantify and qualify: mitochondrial number, endoplasmic reticulum structure, adipose cell size; Gene expression, quantify by microscopy and analyze the protein by western blot.

The study began with 20 women, however, there was withdrawal of 6, ending with 14 women.

Conditions

Conditions: Metabolism Disorder
Mitochondrial Alteration
Physical Activity
Obesity

Keywords:

Study Design

Study Type: Interventional

Primary Purpose: Treatment

Study Phase: N/A

Interventional Study Model: Single Group Assignment

Number of Arms: 1

Masking: None (Open Label)

Allocation: N/A

Enrollment: 14 [Actual]

Arms and Interventions

Arms	Assigned Interventions
Physical Training<br>There was concurrent physical training intervention: strength and aerobic exercises in the same session.<br>Duration: 2 weeks of adaptation and learning to exercise, 8 weeks of physical training.<br>Frequency: 3 times per week Duration: 55 minutes each session. Intensity: 75 to 90% of maximum heart rate.	Physical Training<br>Intervention with concurrent physical training: strength and aerobic exercises in the same session. Duration: 2 weeks of adaptation to physical exercise, 8 weeks of training. Frequency: 3 times a week. Time: 55 minutes each session. Intensity: 75 to 90% of maximum heart rate.

Outcome Measures

Primary Outcome Measure:

1. Changes Body weight
   Body weight was measured by digital balance before and after the intervention

   [Time Frame: Two times: (1) First day and (2) 10 weeks after adaptation and intervention]
2. Changes Body composition
   The change in body composition through deuterium oxide was evaluated.

   [Time Frame: Two times: (1) First day and (2) 10 weeks after adaptation and intervention]
3. Changes White adipose tissue biopsy
   A subcutaneous tissue sample was collected for analysis of: mitochondrial respiration, citrate synthase enzyme, gene expression (UCP2 and UCP3).

   [Time Frame: Two times: (1) First day and (2) 10 weeks after adaptation and intervention]
4. Changes Indirect calorimetry
   With a gas analyzer (indirect calorimeter), we evaluated the metabolic rate and rest (REE) and oxidation of substrates (Lipids and carbohydrates).

   [Time Frame: Two times: (1) First day and (2) 10 weeks after adaptation and intervention]
5. Changes in fatty acids
   Collected in lithium heparin tubes, they were centrifuged.

   [Time Frame: Two times: (1) First day and (2) 10 weeks after adaptation and intervention]
6. Changes oxidative stress
   Collected in lithium heparin tubes, they were centrifuged.

   [Time Frame: Two times: (1) First day and (2) 10 weeks after adaptation and intervention]
7. Changes inflammatory cytokines
   Collected in lithium heparin tubes, they were centrifuged.

   [Time Frame: Two times: (1) First day and (2) 10 weeks after adaptation and intervention]
8. Changes in total cholesterol
   Collected in lithium heparin tubes, they were centrifuged.

   [Time Frame: Two times: (1) First day and (2) 10 weeks after adaptation and intervention]
9. Changes Physical Performance
   Based on the Shuttle Walking Test adaptation.

   [Time Frame: Two times: (1) First day and (2) 10 weeks after adaptation and intervention]

10. Changes in Determination of Lactate
    Blood samples were collected by manual puncture of the earlobe in previously calibrated and heparinized capillary tubes, stored in eppendorf with sodium fluoride. Analyzed by electrochemical lactate analyser.

    [Time Frame: Two times: (1) First day and (2) 10 weeks after adaptation and intervention]

11. Changes Food intake
    Food registry of 3 days, the quantification of the daily intake of nutrients will still be made using software.

    [Time Frame: Two times: (1) First day and (2) 10 weeks after adaptation and intervention]

12. Changes Nitrogen Balance
    Through the collection of urine of 24 hours the dosage of urinary nitrogen will be made by the chemiluminescence method for determination of protein nitrogen.

    [Time Frame: Two times: (1) First day and (2) 10 weeks after adaptation and intervention]

Eligibility

Minimum Age: 20 Years

Maximum Age: 40 Years

Sex: Female

Gender Based:

Accepts Healthy Volunteers: No

Criteria: Inclusion Criteria:

- This study included women with obesity (BMI of 30 to 40 kg / m²), sedentary, with no associated comorbidity, convenience sample

Exclusion Criteria:

- Women who have undergone bariatric surgery, menopause, cancer or any metabolic disease, smokers, alcoholics, who are in use of drugs that act directly on the metabolism and that have medical impediment to the practice of physical exercise.

Contacts/Locations

Central Contact Person:

Central Contact Backup:

Study Officials:

Locations: Brazil
Camila Fernanda Cunha Brandão
Ribeirao Preto, SP, Brazil, 14.048-900
Contact: Camila FC Brandão, Phd student +55 (16) 3315-3375
camilafbrandao@hotmail.com

IPDSharing

Plan to Share IPD: No

References

Citations: BROWN, L.E.; WEIR, J.P.; ASEP procedures recommendation i: accurate assessment of muscular strength and power, Journal of Exercise Physiology, v. 4, n. 3, p. 1-21, 2001.

Burgomaster KA, Howarth KR, Phillips SM, Rakobowchuk M, Macdonald MJ, McGee SL, Gibala MJ. Similar metabolic adaptations during exercise after low volume sprint interval and traditional endurance training in humans. J Physiol. 2008 Jan 1;586(1):151-60. Epub 2007 Nov 8. PubMed 17991697

Chomczynski P, Sacchi N. Single-step method of RNA isolation by acid guanidinium thiocyanate-phenol-chloroform extraction. Anal Biochem. 1987 Apr;162(1):156-9. PubMed 2440339

Colégio Americano de Medicina Esportiva, CAME. Guia para Teste de Esforço e Prescrição de Exercício. 3º Edição, Medsi, Rio de janeiro, RJ, p.25, 1987.

Crump C, Sundquist J, Winkleby MA, Sundquist K. Interactive effects of obesity and physical fitness on risk of ischemic heart disease. Int J Obes (Lond). 2017 Feb;41(2):255-261. doi: 10.1038/ijo.2016.209. Epub 2016 Nov 21. PubMed 27867205

Curtis JM, Grimsrud PA, Wright WS, Xu X, Foncea RE, Graham DW, Brestoff JR, Wiczer BM, Ilkayeva O, Cianflone K, Muoio DE, Arriaga EA, Bernlohr DA. Downregulation of adipose glutathione S-transferase A4 leads to increased protein carbonylation, oxidative stress, and mitochondrial dysfunction. Diabetes. 2010 May;59(5):1132-42. doi: 10.2337/db09-1105. Epub 2010 Feb 11. PubMed 20150287

Daussin FN, Zoll J, Dufour SP, Ponsot E, Lonsdorfer-Wolf E, Doutreleau S, Mettauer B, Piquard F, Geny B, Richard R. Effect of interval versus continuous training on cardiorespiratory and mitochondrial functions: relationship to aerobic performance improvements in sedentary subjects. Am J Physiol Regul Integr Comp Physiol. 2008 Jul;295(1):R264-72. doi: 10.1152/ajpregu.00875.2007. Epub 2008 Apr 16. PubMed 18417645

EIGENTLER, A.; DRAXL, A.; et al. Laboratory Protocol: Citrate synthase a mitochondrial marker enzyme. Mitochondrial Physiology Network, v. 17.04, n. 3, p. 1-11, 2015.

Fernström M, Bakkman L, Loogna P, Rooyackers O, Svensson M, Jakobsson T, Brandt L, Lagerros YT. Improved Muscle Mitochondrial Capacity Following Gastric Bypass Surgery in Obese Subjects. Obes Surg. 2016 Jul;26(7):1391-7. doi: 10.1007/s11695-015-1932-z. PubMed 26471784

Ferreira FC, Bertucci DR, Barbosa MR, Nunes JE, Botero JP, Rodrigues MF, Shiguemoto GE, Santoro V, Verzola AC, Nonaka RO, Verzola RM, Baldissera V, Perez SE. Circuit resistance training in women with normal weight obesity syndrome: body composition, cardiometabolic and echocardiographic parameters, and cardiovascular and skeletal muscle fitness. J Sports Med Phys Fitness. 2017 Jul-Aug;57(7-8):1033-1044. doi: 10.23736/S0022-4707.16.06391-X. Epub 2016 Jul 6. PubMed 27385544

FETT, C.A.; FETT, W.C.R.; MARCHINI, J.S. Fitness Level of Overweight/ Obese Women After 08 Weeks of Aerobic or Mixed Metabolism Exercises. Revista Brasileira de Cineantropometria & Desempenho Humano, v. 11, p. 261-266, 2009.

Foster C. Monitoring training in athletes with reference to overtraining syndrome. Med Sci Sports Exerc. 1998 Jul;30(7):1164-8. PubMed 9662690

Frayn KN. Calculation of substrate oxidation rates in vivo from gaseous exchange. J Appl Physiol Respir Environ Exerc Physiol. 1983 Aug;55(2):628-34. PubMed 6618956

Grimble GK, West MF, Acuti AB, Rees RG, Hunjan MK, Webster JD, Frost PG, Silk DB. Assessment of an automated chemiluminescence nitrogen analyzer for routine use in clinical nutrition. JPEN J Parenter Enteral Nutr. 1988 Jan-Feb;12(1):100-6. PubMed 3125351

Heilbronn LK, Gan SK, Turner N, Campbell LV, Chisholm DJ. Markers of mitochondrial biogenesis and metabolism are lower in overweight and obese insulin-resistant subjects. J Clin Endocrinol Metab. 2007 Apr;92(4):1467-73. Epub 2007 Jan 23. PubMed 17244782

Koh EH, Park JY, Park HS, Jeon MJ, Ryu JW, Kim M, Kim SY, Kim MS, Kim SW, Park IS, Youn JH, Lee KU. Essential role of mitochondrial function in adiponectin synthesis in adipocytes. Diabetes. 2007 Dec;56(12):2973-81. Epub 2007 Sep 7. PubMed 17827403

Kong Z, Sun S, Liu M, Shi Q. Short-Term High-Intensity Interval Training on Body Composition and Blood Glucose in Overweight and Obese Young Women. J Diabetes Res. 2016;2016:4073618. Epub 2016 Sep 28. PubMed 27774458

Kraunsøe R, Boushel R, Hansen CN, Schjerling P, Qvortrup K, Støckel M, Mikines KJ, Dela F. Mitochondrial respiration in subcutaneous and visceral adipose tissue from patients with morbid obesity. J Physiol. 2010 Jun 15;588(Pt 12):2023-32. doi: 10.1113/jphysiol.2009.184754. Epub 2010 Apr 26. Erratum in: J Physiol. 2010 Oct 15; 588(Pt 20):4055. PubMed 20421291

Lepage G, Roy CC. Improved recovery of fatty acid through direct transesterification without prior extraction or purification. J Lipid Res. 1984 Dec 1;25(12):1391-6. PubMed 6530596

Medbø JI, Mamen A, Holt Olsen O, Evertsen F. Examination of four different instruments for measuring blood lactate concentration. Scand J Clin Lab Invest. 2000 Aug;60(5):367-80. PubMed 11003256

Perry CG, Lally J, Holloway GP, Heigenhauser GJ, Bonen A, Spriet LL. Repeated transient mRNA bursts precede increases in transcriptional and mitochondrial proteins during training in human skeletal muscle. J Physiol. 2010 Dec 1;588(Pt 23):4795-810. doi: 10.1113/jphysiol.2010.199448. Epub 2010 Oct 4. PubMed 20921196

Pfrimer K, Moriguti JC, Lima NK, Marchini JS, Ferriolli E. Bioelectrical impedance with different equations versus deuterium oxide dilution method for the inference of body composition in healthy older persons. J Nutr Health Aging. 2012 Feb;16(2):124-7. PubMed 22323345

POLLOCK, M.L.; WILMORE, J.H.; FOX III, S.M. Exercícios na Saúde e na Doença - Avaliação e prescrição para prevenção e reabilitação. Rio de Janeiro: 1986.

Singh SJ, Morgan MD, Scott S, Walters D, Hardman AE. Development of a shuttle walking test of disability in patients with chronic airways obstruction. Thorax. 1992 Dec;47(12):1019-24. PubMed 1494764

Tan S, Wang J, Cao L, Guo Z, Wang Y. Positive effect of exercise training at maximal fat oxidation intensity on body composition and lipid metabolism in overweight middle-aged women. Clin Physiol Funct Imaging. 2016 May;36(3):225-30. doi: 10.1111/cpf.12217. Epub 2014 Nov 19. PubMed 27072372

WEIR JB. New methods for calculating metabolic rate with special reference to protein metabolism. J Physiol. 1949 Aug;109(1-2):1-9. PubMed 15394301

Yin X, Lanza IR, Swain JM, Sarr MG, Nair KS, Jensen MD. Adipocyte mitochondrial function is reduced in human obesity independent of fat cell size. J Clin Endocrinol Metab. 2014 Feb;99(2):E209-16. doi: 10.1210/jc.2013-3042. Epub 2013 Nov 25. PubMed 24276464

Links:

Available IPD/Information:

U.S. National Library of Medicine | U.S. National Institutes of Health | U.S. Department of Health & Human Services
